# Dietary advanced glycation end‐products (dAGEs) are not associated with the risk of cancer incidence. A systematic review and meta‐analysis of prospective cohort studies

**DOI:** 10.1002/fsn3.4396

**Published:** 2024-08-11

**Authors:** Elham Sharifi‐Zahabi, Sepideh Soltani, Fatemeh Hajizadeh‐Sharafabad, Hadi Abdollahzad

**Affiliations:** ^1^ Student Research Committee Kermanshah University of Medical Sciences Kermanshah Iran; ^2^ Yazd Cardiovascular Research Center, Noncommunicable Diseases Research Institute Shahid Sadoughi University of Medical Sciences Yazd Iran; ^3^ Patient Safety Research Center, Clinical Research Institute Urmia University of Medical Sciences Urmia Iran; ^4^ Department of Nutrition, School of Medicine Urmia University of Medical Sciences Urmia Iran

**Keywords:** advanced glycation end‐products, AGEs, cancer, systematic review

## Abstract

A growing body of evidence indicates the association of dietary advanced glycation end‐products (dAGEs) with the risk of cancer. This systematic review and meta‐analysis aimed to assess the overall association between dAGEs and cancer incidence. An extensive search was carried out through online databases including PubMed, Scopus, and Web of Science up to June 2024. All reported HRs and their 95% CIs for risk of cancer were used to estimate log HRs and their standard errors (SEs). The overall risk estimate was obtained using a random effects model. Inter‐study heterogeneity was determined using Cochrane's *Q* test and *I*‐squared. Five prospective cohort studies with a total of 1,220,096 participants and 23,229 incident cancer cases (2193 pancreatic cancers, 11,443 breast cancers, 6162 colorectal cancers, and 3431 total cancers) were included in this meta‐analysis. Compared with the lowest category of dAGEs, the pooled hazard ratio (HR) for overall cancers was 1.04 (95% CI: 0.94, 1.15; *I* ^2^ = 67.9%) for the highest category of dAGEs. Pooled estimates for different types of cancer showed no significant relationship between dAGEs and risk of breast cancer (HR: 1.119; 95% CI: 0.868, 1.444; *I* ^2^ = 77.6%; *N* = 2 studies), pancreatic cancer (HR: 1.242; 95% CI: 0.971, 1.588; *I* ^2^ = 0.0%; *N* = 2 studies), colon cancer (HR: 10.985; 95% CI: 0.887, 1.094; *I* ^2^ = 0.0%; *N* = 2 studies) and rectal cancer (HR: 0.940; 95% CI: 0.616, 1.433; *I* ^2^ = 57.7%; *N* = 2 studies). Dietary AGEs had no significant link with cancer risk. More well‐designed prospective studies are required.

## Graphical abstract

The results of this meta‐analysis revealed that dietary advanced glycation end products (dAGE) levels had no significant association with cancer risk.

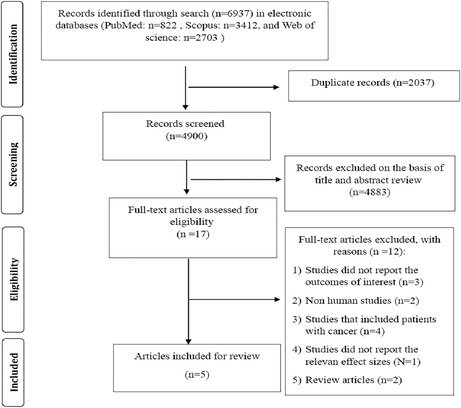

## 1 INTRODUCTION

Advanced glycation end‐products (AGEs, i.e. pentosidine, N‐carboxymethyl‐lysine (CML), and N‐carboxyethyl lysine (CEL)) are complex compounds generated by the nonenzymatic glycation of free amino groups of proteins, lipids, and nucleic acids (Chaudhuri et al., 2018). The circulating AGEs originated from two major sources including endogenous AGEs, produced as a by‐product of sugar metabolism under hyperglycemia and oxidative status, and exogenous AGEs derived from diet, especially highly processed foods (Nagata et al., 2020). Diet‐derived AGEs (dAGEs) are common in highly processed foods and contribute to both circulating and tissue concentration of AGEs (Peterson et al., 2020). A positive association between dAGEs and serum levels of AGEs has been reported (Uribarri et al., 2015). The formation of dAGEs depends on the food preparation and processing steps such as frying, baking, and grilling, in which high temperatures for long periods are used (Turner, 2017; Uribarri et al., 2010). The association of both circulating and dAGEs with metabolic diseases such as atherosclerosis and diabetes has been reported (Chaudhuri et al., 2018; Sharifi‐Zahabi et al., 2021). The deleterious biological consequences of AGEs are augmented through their direct impacts on tissues or interaction with their membrane receptor (RAGE), leading to the induction of oxidative stress and several inflammatory signaling cascades (Ott et al., 2014; Prasad & Mishra, 2018). Oxidative stress caused by AGEs may have a crucial role in the development of cancer‐associated DNA damage (Turner, 2017). Moreover, an increasing amount of evidence indicates the association of AGEs with the risk of cancer. Increased circulating levels of AGEs in women with breast cancer compared to healthy women have been reported (Tesarova et al., 2007; Walter et al., 2019). Also, AGEs may have a differential impact on subtypes of breast cancer depending on hormone receptor status (Nass et al., 2017; Walter et al., 2019). Additionally, AGEs can promote the proliferation, migration, and invasion of cancer cell lines. Hence, dAGEs, a prominent source of exogenous AGEs, may have a role in the development of cancer (Riehl et al., 2009; Sharaf et al., 2015). Moreover, dAGEs have also been reported to be associated with colorectal cancer progression, mainly owing to their in vitro capability to increase tumor cell proliferation (Geicu et al., 2020). However, some other studies have reported conflicting results. In the European Prospective Investigation into Cancer and Nutrition (EPIC) study, conducted on participants from 10 European countries, dAGEs were negatively correlated with the risk of hepatocellular and colorectal carcinoma, whereas it positively related to the increased risk of gallbladder cancer (Aglago et al., 2021; Mayén et al., 2021). Based on previous equivocal findings we aimed to conduct a systematic review and meta‐analysis to quantitatively measure the relationship between dAGEs and cancer incidence. This meta‐analysis is the first one on the association between dietary AGEs and cancer.

## 2 METHODS

The current systematic review and meta‐analysis adhered to the recommendations set by the Preferred Reporting Items for Systematic Reviews and Meta‐Analysis (PRISMA) statement. The protocol of this study was registered in the International Prospective Register of Systematic Reviews (PROSPERO) with the registration ID: CRD42024559403.

### 2.1 Search strategy

A combination of “AGE” and “cancer” related MeSH (Medical Subject Headings) and non‐MeSH terms were applied to retrieve related papers by means of the online databases, including PubMed, Scopus, and Web of Science, up to 20 June 2024 **(**Table S1 **)**. Furthermore, the initial 4 pages of Google Scholar and the bibliography of included studies were inspected manually to find other, potentially related, studies. The search processes were unconstrained in terms of language or time. Two reviewers individually assessed the results of the document search to determine the relevant papers. Any disput was settled through consultation with the corresponding author (HA).

### 2.2 Study selection

The EndNote software version 20.4.1 was used to manage the retrieved articles. The eligibility of each study was assessed by two reviewers (ESZ and FH) independently and according to the inclusion criteria. The inclusion criteria for this study were in accordance with the PECOS scheme (Morgan et al., 2018): the population was subjects aged >18 years old; the exposures were dAGEs; the comparator was the topmost versus bottommost intake of dAGEs; the outcome was cancer incidence; the setting included all cohort studies (prospective, retrospective, nested case–control and case‐cohort) in which odds ratios (ORs), or risk ratios (RRs) or hazard ratios (HRs) with their 95% confidence intervals were used to report the associations of dAGEs with cancer incidence. The minimum follow‐up duration for outcomes was considered to be 1 year.

We omitted the letters, comments, reviews, ecological studies, randomized controlled trials, case reports, and cross‐sectional and case–control studies. Moreover, studies conducted on children or teenagers, patients with CVD, and pregnant women were also excluded. Excluded articles were confirmed by the two reviewers and any disparity were reconciled by discussion with the principal investigator (HA).

### 2.3 Data extraction

The full texts of identified studies were reviewed by pair of researchers (ESZ and FH). All requisite data was individually retrieved by one author, based on a prearranged screening instrument on Excel, and checked by another author. Any disparity was settled through guidance from the principal researcher. Extracted information for each qualifying study comprised the following items: the last name of the first researcher, year of publication, country/region, study design, duration of follow‐up, participant's characteristics, mean or range of age, gender, total participants, number of cancer cases, exposure, the method used for exposure assessment, outcome, any adjustment for confounding variables, relevant effect sizes for the topmost versus the botommost category of exposure and study quality score. In the studies where multiple effect measures were reported for the risk of cancer, we chose the effect measures in a fully adjusted model. If a study had multiple follow‐up durations, we considered the outcome related to the longest duration for inclusion in the analysis.

### 2.4 Quality assessment of the studies

The studies were appraised for quality using the Risk Of Bias In Non‐randomized Studies of Exposure effects (ROBINS‐E) (Higgins et al., 2024). This checklist comprises seven key segments appraising bias due to (1) confounding variables, (2) selection of participants, (3) exposure assessment, (4) misclassification during follow‐up, (5) missing data, (6) measurement of the outcome, (7) selective reporting of the results.

### 2.5 Quality of meta‐evidence

The Grading of Recommendations, Assessment, Development, and Evaluations (GRADE) approach was used to assess the quality of the evidence for each association (Guyatt et al., 2011). GRADE rates the certainty of evidence as high, moderate, low, or very low. Table S2 provides descriptions of the domains of the GRADE tool and how to judge each domain. Two authors independently performed GRADE assessments and disagreements were solved by consensus. The final decision of quality evidence was assessed with the use of GRADE pro software (Grade Working Group).

### 2.6 Statistical analysis

All reported HRs and their 95% CIs for risk of cancer were utilized to estimate log HRs and their standard errors (SEs). A random effects model which considers inter‐study heterogeneity was applied to estimate the overall effect measure (DerSimonian & Laird, 1986). Heterogeneity among studies was explored by applying Cochrane's *Q* test and *I*‐squared (Higgins et al., 2003).

To establish a harmonized and consistent methodology in the meta‐analysis, all effect sizes for the relationship between dAGEs and cancer were changed to the upper versus lower quintile of dAGEs attribution pattern in each individual study (Chêne & Thompson, 1996). For those studies that provided the risk of cancer in quintiles of dAGEs, the HRs or RRs were employed in the analysis as reported. In those studies that classified the values of exposure in quartiles, we standardized the HRs or RRs provided by the primary studies to measures that represented the comparison between the first and bottom quintile of dAGEs attribution using defined methods (Chêne & Thompson, 1996). According to the mentioned method, we required a conversion factor to convert the log RRs from the reported effect sizes to the upper versus lower quintile. This conversion factor was determined by calculating the ratio of anticipated differences in average measures of the standardized exposure. For instance, a factor of 2.54 represented the disparity between the average of the topmost and bottom quartiles. While the anticipated difference in averages of the higher and bottom quintiles of the standard normal attribution was 2.8. Hence, via multiplying the conversion factor of 2.8/2.54 by the logarithm of the RR (logRR) and its related standard error, we were able to transform a comparison between the topmost versus bottommost quartile into an equivalent comparison between the topmost versus bottommost quintile. In studies where the results were reported for men and women separately, effect sizes related to both groups were initially combined using the fixed model and then integrated into the main analysis. As the number of primary studies was less than the minimum needed 10, publication bias was not assessed (Higgins & Green, 2008). Due to the small number of the included studies, subgroup analysis was not performed. However, we extracted the effect sizes related to the different cancer types in each study and conducted a meta‐analysis on those studies with the same outcomes. Statistical analyses were done in Stata, version 17 (Stata Crop). Statistical significance was attributed to values of <0.05.

### 2.7 Sensitivity analysis

A sensitivity analysis was done, by systematically deleting individual studies from the analysis (Cro et al., 2016). Besides, various analyses were conducted on the studies that, reported a similar type of cancer (breast cancer (Omofuma et al., 2020; Peterson et al., 2020), colon cancer (Aglago et al., 2021; Wada et al., 2022), rectal cancer (Aglago et al., 2021; Wada et al., 2022), and pancreatic cancer (Jiao et al., 2015; Wada et al., 2022)), had the same location (Jiao et al., 2015; Omofuma et al., 2020; Peterson et al., 2020) (USA), and adjusted for confounders (dietary meat and meat product (Aglago et al., 2021; Jiao et al., 2015; Omofuma et al., 2020; Peterson et al., 2020), dietary fat (Jiao et al., 2015; Omofuma et al., 2020; Peterson et al., 2020), alcohol consumption (Jiao et al., 2015; Omofuma et al., 2020; Peterson et al., 2020; Wada et al., 2022), and physical activity (Aglago et al., 2021; Omofuma et al., 2020; Peterson et al., 2020; Wada et al., 2022)) to recognize if the overall effect measure was influenced by any of these factors.

## 3 RESULTS

### 3.1 Literature search

**FIGURE 1:**
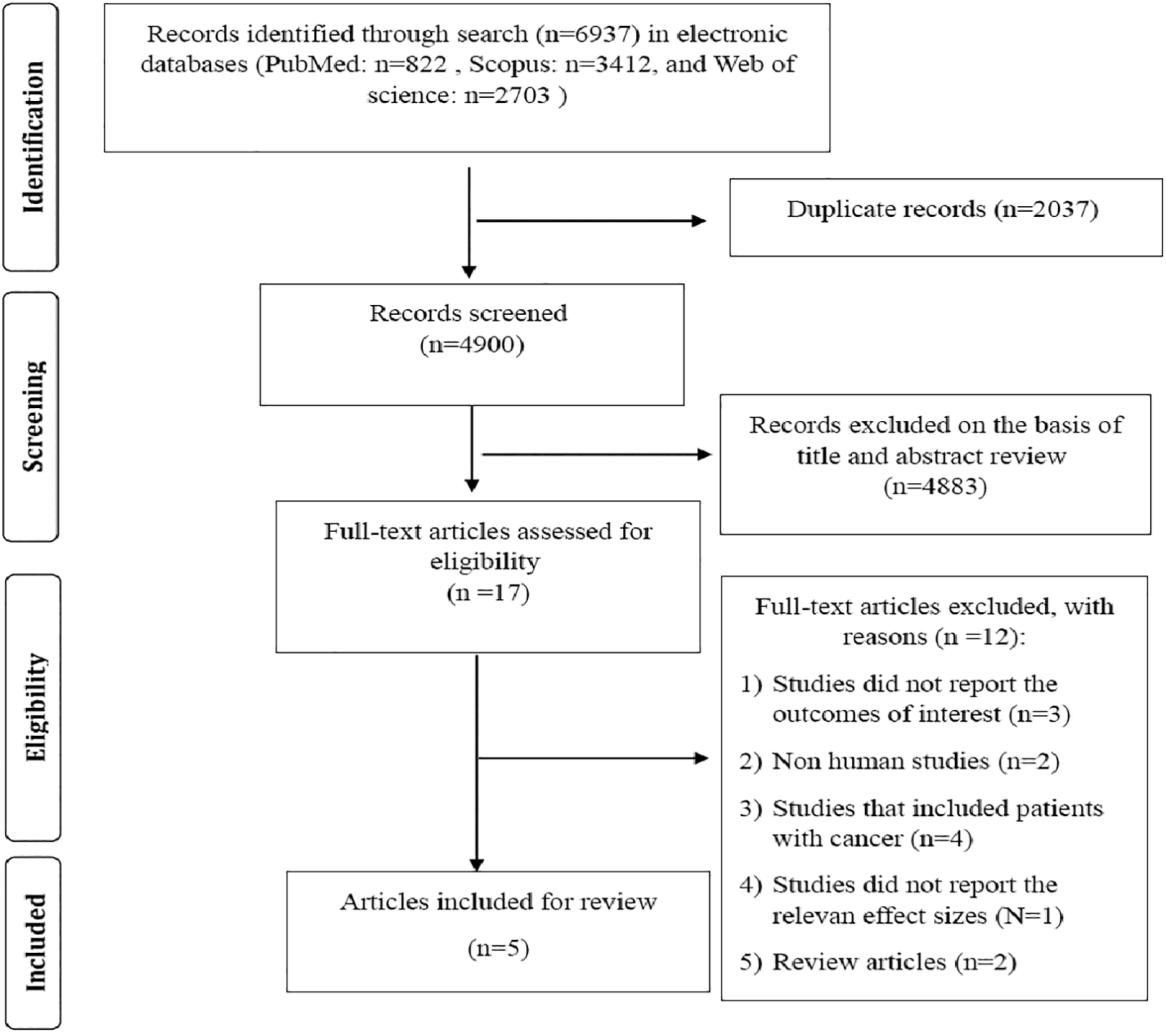
Flow diagram of the literature search and study selection process.

The detailed literature search is depicted in Figure 1. In the primary search, 6937 articles were discovered. After eliminating duplicate publications, an additional 4884 articles were removed based on reviewing their titles and related abstracts. A total of 16 papers were reviewed in full text; three of them failed to report cancer incidence (Ebert et al., 2020; Hosseini et al., 2023; Si et al., 2024), two were non‐human studies (Abe & Yamagishi, 2008; Kuniyasu et al., 2003), four included patients with cancer (Jahromi et al., 2023; Kong et al., 2015; Mao et al., 2021; Omofuma et al., 2021), One did not report the relevant effect sizes (Mayén et al., 2021) and two were review studies (Peterson & Ligibel, 2024; Takino et al., 2015), so they were not included in the review. Finally, five articles involving 1,220,096 participants and 23,229 incident cancer cases (2193 pancreatic cancers, 11,443 breast cancers, 6162 colorectal cancers, and 3431 total cancers) were eligible to be included in the overall analysis.

### 3.2 Study characteristics

Table 1 provides an overview of the studies' characteristics. All studies had prospective cohort design with a minimum follow‐up period of 10 years [10–13.5 years]. Three studies took place in the United States, one in European countries, and another in Japan. The average age of the participants varied from 51 to 62 years. Two studies reported separate relative risks for men and women. Dietary AGEs were assessed with a food frequency questionnaire. Cancer incident was mostly ascertained from national cancer registries. The dietary content of CML was considered as an approximate estimation of AGEs. Moreover, Age, sex, BMI, and smoking were confounding factors adjusted in all studies. However, some studies did not adjust for other confounding variables including physical activity (Jiao et al., 2015), meat or meat products (Aglago et al., 2021), dietary fat (Aglago et al., 2021; Wada et al., 2022), and alcohol consumption (Aglago et al., 2021). As, the subtypes of cancer were evaluated in two studies by (Wada et al., 2022) (colon cancer, breast cancer, and pancreatic cancer) and (Aglago et al., 2021) (colon cancer and rectal cancer) these studies were included in the sensitivity analysis to explore the source of heterogeneity.

**TABLE 1:** Main characteristics of studies examining the association between dietary advanced glycation end products and cancer incidence.

First author/ year	Region	Study design/name	Follow‐up (year)	Population	Age (year) (mean or range)	Sex	Participants/cases	Exposure/exposure assessment	Outcome	Adjustment	Comparison	Quality score
Aglago et al. (2021)	European Countries	Cohort/ EPIC Study	13	Participants from 10 European countries (Denmark, France, Germany, Greece, Italy, the Netherlands, Norway, Spain, Sweden, and the United Kingdom)	51	M/F	450,111/ 6162	CML /FFQ CEL /FFQ MG‐H1 /FFQ UPLC–MS/ MS method	Colorectal cancer (colon cancer, rectal cancer)	Age, sex, and center; BMI, height, education, physical activity, smoking, and energy intake; Mediterranean diet score	Q5 Versus Q1	Serious
Jiao et al. (2015)	USA	Cohorts/NIH‐AARP Diet and Health Study	10.5	U.S. Population	62	M F	310,458/ 1407 217,793/786	CML /FFQ ELISA method	Pancreatic cancer	Age (continuous). race, education, diabetes, smoking status, first degree family history of cancer, BMI, and alcohol, calories, carbohydrate intake, and calcium, and red meat intake on a continuous scale.	Q5 Versus Q1	Serious
Omofuma et al., (2020)	USA	Cohort/PLCO cohort/Cancer Screening Trial	11.5	U.S. Population	62	F	27,464/1592	CML/FFQ ELISA method	Breast cancer	Age, energy intake, alcohol, BMI, vigorous activity, race, marital status, education, study center, smoking status, family history, age at menarche, age at menopause, age at first birth, no. of live birth, PMH use, OC use, oophorectomy, hysterectomy and dietary intake of total fat and red meat	Q5 Versus Q1	Moderate
Peterson et al., (2020)	USA	Cohort/Health‐AARP Diet and Health Study	12.8	Postmenopausal women	62	F	183,548/9851	CML/FFQ ELISA method	Breast cancer ELISA method	Age at study entry, race, education, body mass index, age at menarche, age at first birth, age at menopause, physical activity smoking, alcohol intake, use of hormone‐replacement therapy, family history of breast cancer, history of breast biopsy and total energy intake, total meat and fat intake.	Q5 Versus Q1	Moderate
Wada et al., (2022)	Japan	Cohort/Takayama study	12.3	Japenese men/ women	55	M F	14,173/1954 16,549/1477	CML/FFQ UPLC–MS/ MS. method	Total cancer (colon cancer, pancreatic cancer, rectal cancer and..)	Age, body mass index, education years, history of diabetes, physical activity score (METs h/week), smoking status , alcohol consumption (g/day), and intakes of total energy (kcal/day).salt intake, height, aspirin use and intakes of red meat and processed meat (g/d), coffee consumption	Q4 Versus Q1	Moderate

### 3.3 Quality assessment

A detailed quality assessment for each separate study has been presented in Table 2. Two studies (Aglago et al., 2021; Jiao et al., 2015) had a serious risk of bias and the remaining had a moderated risk of bias. Three studies moderately adjusted for confounding variables(age, sex, alcohol, smoking, and dietary intakes) (Omofuma et al., 2020; Peterson et al., 2020; Wada et al., 2022), and the other two studies did not fully control for potential confounders (Aglago et al., 2021; Jiao et al., 2015). Two studies had a low risk of bias for exposure assessment and misclassification during follow‐up (Aglago et al., 2021; Wada et al., 2022). Four studies were identified as having a low risk of bias for measuring the outcome because cancer incidence was ascertained from cancer registries (Aglago et al., 2021; Jiao et al., 2015; Wada et al., 2022). None of the studies were biased by the selection of participants, missing data, and selective reporting of the results.

**TABLE 2:** Quality assessment based on ROBINS‐E judgment for each domain and overall.

Study	Bias due to confounding	Bias due to the selection of participants	Bias due to exposure assessment	Bias due to misclassification during follow‐up	Bias due to missing data	Bias due to measurement of the outcome	Bias due to selective reporting of the results	Overall judgment
Aglago et al., (2021)	Serious	Low	Low	Moderate	Low	Low	Low	Serious
Jiao et al., (2015)	Serious	Low	Moderate	Low	Low	Low	Low	Serious
Omofuma et al., (2021)	Moderate	Low	Moderate	Moderate	Low	Moderate	Low	Moderate
Peterson et al., (2020)	Moderate	Low	Moderate	Moderate	Low	Low	Low	Moderate
Wada et al., (2022)	Moderate	Low	Low	Low	Low	Low	Low	Moderate

### 3.4 Certainty of evidence

TABLE SS2 shows the certainty of evidence by the GRADE approach. The certainty of evidence was rated as “Low” due to “very serious” risk of bias, since, two studies which included 80% of the total participants, were rated as having a serious risk of bias (Aglago et al., 2021; Jiao et al., 2015).

### 3.5 Meta‐analysis

#### 3.5.1 Dietary AGEs and cancer incidence

**FIGURE 2:**
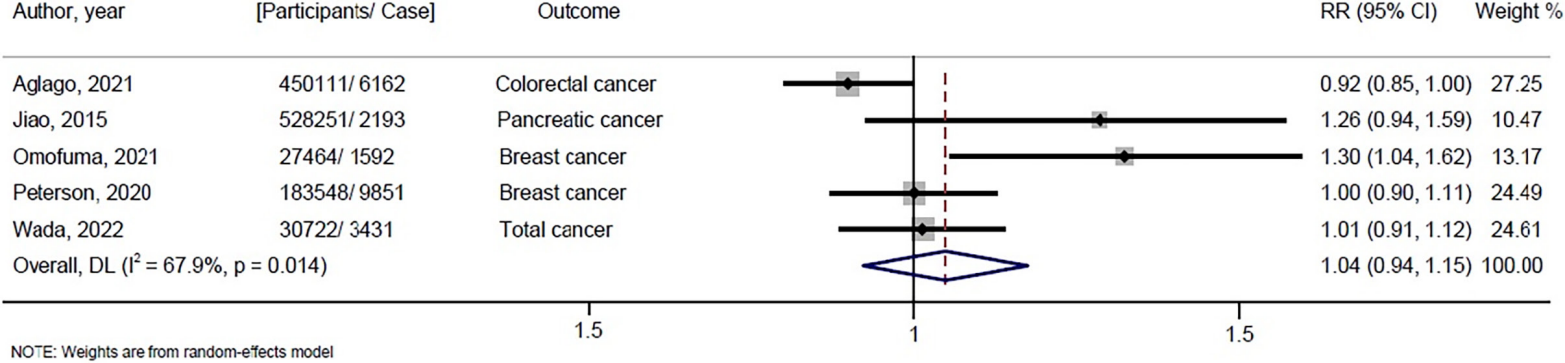
Forest plot of the association between dietary AGEs and cancer risk.

Five studies included in the meta‐analysis investigated the link between dietary AGEs and the risk of overall and different cancer types. Compared with the lowest dietary AGEs, the pooled HR for overall cancer was 1.04 (95% CI: 0.94, 1.15; *I* ^2^ = 67.9%) for the fifth highest category. Figure 2 shows the findings according to cancer type. We refrained from doing a subgroup analysis due to the limited number of available studies. However, pooled estimates for different type of cancer showed no significant relationship between dAGEs and risk of breast cancer (HR; 1.119; 95% CI: 0.868, 1.444; *I* ^2^ = 77.6%; *N* = 2 studies), pancreatic cancer (HR, 1.242; 95% CI: 0.971, 1.588; *I* ^2^ = 0.0%; *N* = 2studies), colon cancer (HR: 10.985; 95% CI: 0.887, 1.094; *I* ^2^ = 0.0%; *N* = 2 studies) and rectal cancer (HR: 0.940; 95% CI: 0.616, 1.433; *I* ^2^ = 57.7%; *N* = 2 studies).

#### 3.5.2 Sensitivity analysis

The stability of the pooled results was further confirmed by eliminating one study sequential in the sensitivity analysis. Additionally, the results of sensitivity analysis based on the reported cancer type, study location (US country), and any adjustments for confounders, indicated that none of these variables affected the results of the overall analysis (Table 3).

**TABLE 3:** Sensitivity Analysis.

First author/ year	Studies	Effect size	*I* ^2^
Cancer type			
Breast cancer	Omofuma et al. (2020) and Peterson et al., (2020)	1.119 (95% CI: 0.868, 1.444)	77.6%
Pancreatic cancer	Jiao et al. (2015) and Wada et al. (2022)	1.242 (95% CI: 0.971, 1.588)	0.0%
Colon cancer	Aglago et al. (2021) andWada et al. (2022)	10.985 (95%CI: 0.887, 1.094)	0.0%
Rectal cancer	Aglago et al. (2021) andWada et al. (2022)	0.940 (95% CI: 0.616, 1.433)	57.7%
Location			
USA	Jiao et al. (2015), Omofuma et al. (2020), Peterson et al. (2020)	1.150 (95% CI: 0.948, 1.394)	67.2%
Adjustment			
Dietary meat and meat product	Jiao et al. (2015), Omofuma et al. (2020), Peterson et al. (2020), Wada et al. (2022)	1.084 (95% CI: 0.966, 1.217)	56.1%
Dietary fat	Jiao et al. (2015), Omofuma et al. (2020), Peterson et al. (2020)	1.150 (95% CI: 0.948, 1.394)	67.2%
Alcohol consumption	Jiao et al. (2015), Omofuma et al. (2020), Peterson et al. (2020), Wada et al. (2022)	1.084 (95% CI: 0.966, 1.217)	56.1%
Physical activity	Aglago et al. (2021), Omofuma et al. (2020), Peterson et al. (2020), Wada et al. (2022)	1.012 (95% CI: 0.916, 1.119)	67.2%

USA, Unites States of America.

## 4 DISCUSSION

Based on the knowledge available to us, this study is the first one that systematically assessed the existing findings on the association of dietary AGE, mainly CML, with the risk of cancer. CML has been known as the main product commonly used for dAGEs estimation since it appears to be among the most abundant and chemically well‐studied AGEs in the body. Unlike primary findings supporting a link between dAGE and cancer (Peterson et al., 2020; Walter et al., 2019), we observed no relationship between the levels of consumed dAGE and the incidence of cancer.

As previously mentioned a direct link between diet‐derived and circulating AGE has been reported (Uribarri et al., 2015). The association between increased AGE accumulation and risk of cancer has also been highlighted in recent *invivo* studies (Kang et al., 2012; Menini et al., 2018; Takino et al., 2015). All these studies indicated the enhanced susceptibility to cancer initiation and development via increasing AGE/RAGE‐dependent stress response, which can subsequently lead to enhanced oxidative stress and long‐term inflammation. In other words, RAGE signaling serves as a vital connection between the accumulated AGEs and cancer via its ability to stimulate the NF‐κB pathway (Schröter & Höhn, 2018). Overall, a growing body of evidence demonstrated that AGEs, through engaging with RAGE, could exhibit a crucial role in various forms of cancer and participate in tumor development, migration, and metastasis (Schröter & Höhn, 2018). Contrary to the ample evidence supporting the impact of AGEs on cancer progress, human studies in this regard are controversial. To date, six cohort studies have focused on the relationship between dAGEs and cancer risk (Aglago et al., 2021; Jiao et al., 2015; Mayén et al., 2021; Omofuma et al., 2020; Peterson et al., 2020; Wada et al., 2022). The overall findings of the present work are in conflict with the recenr research of Wada et al. (2022) who reported no statistically significant relationship between the high amount of CML intake and the risk of cancer incidence in male and female subjects. By contrast, in a study, (Jiao et al., 2015), reported that dietary intake of CML had a positive and significant association with the risk of pancreatic cancer in males. Moreover, a positive link between CML consumption and postmenopausal breast cancer has been reported (Peterson et al., 2020). In the Prostate, Lung, Colorectal, and Ovarian Cancer Screening Trial, higher intake of CML was also associated with a higher risk of breast cancer (Omofuma et al., 2020). In the area of AGE and its linked inflammatory diseases, the role of other contributing agents should not be overlooked. A circulating variant of RAGE, namely soluble RAGE (sRAGE) is secreted from cells and prevents the deleterious effects of RAGE‐signaling by binding to its ligands. Therefore it can represent a natural inhibitor of RAGE‐AGEs interaction. Moreover, sRAGE values may be considered a beneficial biofactor for assessing inflammatory disorders such as cancer. Since, not only does it help evaluate the seriousness of the diseases, but it also aids in examining the therapeutic response (Tesarova et al., 2015). Hence, assessing this biomarker along with dAGEs might provide better insight into the association of dAGEs with cancer development. However none of the included studies considered this biomarker in their analysis. Although all the included studies adjusted for age, gender, BMI, and smoking status, some studies did not control for other possible confounding factors such as physical activity (Jiao et al., 2015), alcohol consumption (Aglago et al., 2021), dietary fat (Aglago et al., 2021; Jiao et al., 2015; Wada et al., 2022), carbohydrate (Omofuma et al., 2020; Peterson et al., 2020; Wada et al., 2022), and meat intake (Aglago et al., 2021). These issues led to a reduction in the quality of the included studies with nearly 40% of them facing a serious risk of bias based on ROBINS‐E checklist. It is worth mentioning that, the small number of the included studies did not allow us to conduct a subgroup analysis based on the type of adjustment for confounders, to find the source of heterogeneity. However, the results of sensitivity analysis, revealed that removing those studies that did not adjust for a specific confounder had no significant effect on the overall results. Additionally, applying reliable analytical methods to estimate dietary AGE levels would facilitate the interpretation of the findings (Schröter & Höhn, 2018). It should be noted that three of the five included studies (Jiao et al., 2015; Omofuma et al., 2020; Peterson et al., 2020) assessed the amount of CML in foods via a database that was based on an ELISA and reported positive relationship between dietary CML and the risk of cancer. However, it should not be ignored, that the accuracy and reliability of the ELISA technique for estimation of CML has been reported to be questionable. It seems that the ELISA method overestimates the CML values for high‐fat foods such as butter, olive oil, and mayonnaise and underestimates its values for foods with highe amounts of carbohydrates like cereals, biscuits, and cookies in comparison with a more precise method, called UPLC–MS/ MS (Nowotny et al., 2018; Omofuma et al., 2020). Conversely, in other studies, dietary CML was estimated via databases based on UPLC–MS/MS (Aglago et al., 2021; Wada et al., 2022). The findings of these studies revealed that dietary CML was negatively associated with the risk of colorectal cancer, and positively linked with hepatocellular cancer in men (Aglago et al., 2021; Wada et al., 2022). Therefore it appears that heterogeneity in methods used to estimate dAGEs may also contribute to our nonsignificant results. Moreover, heterogeneity in cancer sites might be another contributing factor to our non‐significant results. In Wada et al. study (2022) dAGE had a positive association with liver cancer and a negative association with stomach cancer. This inverse association might be attributed to the effect of the gut microbiome on the metabolizing of dAGEs, such that the yielded compounds may not be recognized by RAGE and not induce an inflammatory reaction within the gut (Bui et al., 2019; Hellwig et al., 2015, 2019). However, the results of the sensitivity analysis based on cancer sites showed no significant association between dAGEs and the risk of breast cancer, pancreatic cancer, colon cancer, and rectal cancer. As previously mentioned, the results of GRADE analysis indicated a low certainty of evidence. This factor may also contribute to the null results of this meta‐analysis. Thus, the findings should be interpreted with caution.

A rigorous systematic search in online databases, applying an extensive and complete methodology to retrieve available studies, assessing the certainty of evidence by GRADE, and examining the influence of specific studies on the overall effect measure by sensitivity analysis are some strengths of the current study. Conversely, the current study faced some limitations that could undermine our findings. As we conducted a meta‐analysis of observational studies residual or unmeasured confounding variables might affect the magnitude of the association between dAGEs and cancer risk. While all the studies controlled for potential confounders, (such as age, sex, BMI, and smoking) some did not consider the dietary intake of other possibly relevant nutrients such as the quantity and type of dietary fats, carbohydrates, comorbidity status, and medication use, thus introducing a risk of bias. Additionally, measurement errors in dietary assessment may affect the associations between dAGE and cancer. Finally, the small number of included studies limited the efficiency of conducting any subgroup analyses and assessing publication bias.

## 5 CONCLUSION

The outcome of this meta‐analysis demonstrated that dAGE levels had no significant relationship with the risk of cancer incidence. Considering positive associations between dAGEs and the incidence of cancer reported by some primary studies, and due to the limited number of available articles in this field, cancer survivors are recommended to limit consumption of highly processed food till obtaining more solid evidence.

## AUTHOR CONTRIBUTIONS

**Elham Sharifi‐Zahabi:** Conceptualization (equal); data curation (equal); methodology (equal); writing – original draft (equal). **Sepideh Soltani:** Formal analysis (equal). **Fatemeh Hajizadeh‐Sharafabad:** Conceptualization (equal); data curation (equal); methodology (equal). **Hadi Abdollahzad:** Supervision (equal); writing – review and editing (equal).

## FUNDING INFORMATION

No funding was received.

## CONFLICT OF INTEREST STATEMENT

The authors declare no conflicts of interest.

## Supporting information


Table S1.

Table S2.

## Data Availability

The dataset applied and analyzed for the present study is available from the corresponding author on a reasonable request.
