# Supplementary material for: Dietary advanced glycation end‐products (dAGEs) are not associated with the risk of cancer incidence. A systematic review and meta‐analysis of prospective cohort studies
Source: Food Sci Nutr. 2024 Aug 11;12(10):7788–97. doi: 10.1002/fsn3.4396 (PMC11521677; doi:10.1002/fsn3.4396)
Supplement: Supplementary file 1 — Table S1. [file FSN3-12-7788-s001.docx]

**Dietary Advanced Glycation End-products (dAGEs) Are not Associated with the Risk of Cancer Incidence. A Systematic Review and Meta-analysis of Prospective Cohort Studies**

**Elham Sharifi-Zahabi**

**Supplementary Table 1: Search strategy**

| **Web of Science**  **Up to 6/20/2024** | (TS=("Maillard Reaction End Products") OR TS=("Maillard Reaction Products") OR TS=("Maillard Reaction") OR TS=("Advanced Maillard Reaction End Products") OR TS=(carboxymethyllysine) OR TS=("carboxymethyl-lysine") OR TS=(pentosidine) OR TS=(dicarbonyl) OR TS=(carboxyethyllysine) OR TS=(amadoriproduct) OR TS=(pyrraline) OR TS=(methylglyoxal) OR TS=("Glycation End products") OR TS=("Advanced Glycation") OR TS=("Glycosylation End Products") OR TS=("Advanced Glycosylation End products") OR TS=("Advanced Glycosylation") OR TS=(Glycosylation End products) OR TS=("Advanced Glycation End Products") OR TS=("Advanced Glycosylation End Products") OR TS=("Maillard Products") OR TS=("Advanced Glycation End products") OR TS=("Advanced Glycosylation End-Product") OR TS=("Advanced Glycosylation End Product") OR TS=("Advanced Glycosylation End-Products") OR TS=("Advanced Glycosylation End Products")) AND (TS=(cancer) or TS=(carcinoma) or TS=(malignancy)) .N=2703 |
| --- | --- |
| **Scopus** | **(**TITLE-ABS-KEY **("**Maillard Reaction End Products") **OR** TITLE-ABS-KEY **("**Maillard Reaction Products") **OR** TITLE-ABS-KEY **("**Advanced Maillard Reaction End Products") **OR** TITLE-ABS-KEY **(**carboxymethyllysine) **OR** TITLE-ABS-KEY **("**carboxymethyl-lysine") **OR** TITLE-ABS-KEY **(**pentosidine) **OR** TITLE-ABS-KEY **(**dicarbonyl) **OR** TITLE-ABS-KEY **(**carboxyethyllysine) **OR** TITLE-ABS-KEY **(**amadoriproduct) **OR** TITLE-ABS-KEY (pyrraline) OR TITLE-ABS-KEY (methylglyoxal) **OR** TITLE-ABS-KEY ("Glycation End products") OR TITLE-ABS-KEY ("Advanced Glycation") **OR** TITLE-ABS-KEY **("**Glycosylation End Products") **OR** TITLE-ABS-KEY **("**Advanced Glycosylation End products") OR TITLE-ABS-KEY ("Advanced Glycosylation") **OR** TITLE-ABS-KEY **(**Glycosylation End products) **OR** TITLE-ABS-KEY **("**Advanced Glycation End Products") **OR** TITLE-ABS-KEY **("**Maillard Products") **OR** TITLE-ABS-KEY ("Advanced Glycosylation End-Product") **OR** TITLE-ABS-KEY ("Advanced Glycosylation End Product") OR TITLE-ABS-KEY ("Advanced Glycosylation End-Products")**)** AND (TITLE-ABS-KEY (cancer) OR TITLE-ABS-KEY (malignancy) OR TITLE-ABS-KEY (carcinoma)) . N=3412 |
| **PUBMED** | **(**Maillard Reaction End Products **[MeSH Terms] OR** Maillard Reaction End Products **[Title/Abstract] OR** Maillard Reaction Products **[MeSH Terms] OR** Maillard Reaction Products **[Title/Abstract] OR** Products, Maillard Reaction **[MeSH Terms] OR** Products, Maillard Reaction **[Title/Abstract] OR** Reaction Products, Maillard **[MeSH Terms] OR** Reaction Products, Maillard **[Title/Abstract] OR** Advanced Maillard Reaction End Products **[MeSH Terms] OR** Advanced Maillard Reaction End Products**[Title/Abstract] OR** carboxymethyllysine**[MeSH Terms] OR** carboxymethyllysine**[Title/Abstract] OR** carboxymethyl-lysine**[MeSH Terms] OR** carboxymethyl-lysine**[Title/Abstract] OR** pentosidine**[MeSH Terms] OR** pentosidine**[Title/Abstract] OR** dicarbonyl**[MeSH Terms] OR** dicarbonyl**[Title/Abstract] OR** carboxyethyllysine**[MeSH Terms] OR** carboxyethyllysine**[Title/Abstract] OR** amadoriproduct **[MeSH Terms]** OR amadoriproduct**[Title/Abstract] OR** pyrraline**[MeSH Terms]** pyrraline **[Title/Abstract]** OR methylglyoxal**[MeSH Terms] OR** methylglyoxal**[Title/Abstract] OR** Glycation Endproducts, Advanced**[MeSH Terms] OR** Glycation Endproducts, Advanced **[Title/Abstract] OR** Endproducts, Advanced Glycation**[MeSH Terms] OR** Endproducts, Advanced Glycation **[Title/Abstract] OR** Glycosylation End Products, Advanced **[MeSH Terms] OR** Glycosylation End Products, Advanced**[Title/Abstract] OR** Advanced Glycosylation Endproducts**[MeSH Terms] OR** Advanced Glycosylation Endproducts**[Title/Abstract] OR** Endproducts, Advanced Glycosylation**[MeSH Terms] OR** Endproducts, Advanced Glycosylation**[Title/Abstract] OR** Glycosylation Endproducts, Advanced**[MeSH Terms] OR** Glycosylation Endproducts, Advanced**[Title/Abstract] OR** Advanced Glycation End Products**[MeSH Terms] OR** Advanced Glycation End Products**[Title/Abstract] OR** Advanced Glycosylation End Products**[MeSH Terms] OR** Advanced Glycosylation End Products**[Title/Abstract] OR** Maillard Products**[MeSH Terms] OR** Maillard Products**[Title/Abstract] OR** Products, Maillard**[MeSH Terms] OR** Products, Maillard**[Title/Abstract] OR** Advanced Glycation Endproducts**[MeSH Terms] OR** Advanced Glycation Endproducts**[Title/Abstract] OR** AGE**[MeSH Terms] OR** AGE**[Title/Abstract] )** AND (cancer **[Title/Abstract]** OR cancer **[MeSH Terms] OR** carcinoma **[Title/Abstract] OR** carcinoma **[MeSH Terms] OR** malignancy **[Title/Abstract]** OR malignancy **[MeSH Terms]**) . N= 822 |

Studies that failed to report cancer incidence

E. Hosseini, Z. Mokhtari, H. Poustchi, et al., Dietary Advanced Glycation End Products and Risk of Overall and Cause-Specific Mortality: Results from the Golestan Cohort Study, *International Journal of Environmental Research and Public Health*, 2023, **20**.

C. Si, F. Liu, Y. Peng, et al., Association of total and different food-derived advanced glycation end-products with risks of all-cause and cause-specific mortality, *Food and Function*, 2024, **15**, 1553-1561.

H. Ebert, M. E. Lacruz, A. Kluttig, et al., Association between advanced glycation end products, their soluble receptor, and mortality in the general population: results from the CARLA study, *Experimental Gerontology*, 2020, **131**, 110815.

Non human studies

R. Abe and S. Yamagishi. AGE-RAGE system and carcinogenesis, *Current Pharmaceutical Design*, 2008, **14**, 940-945.

H. Kuniyasu, Y. Chihara and H. Kondo. Differential effects between amphoterin and advanced glycation end products on colon cancer cells, *International journal of cancer*, 2003, **104**, 722-727.

Studies that included patients with cancer

M. K. Jahromi, A. N. Tehrani, H. Farhadnejad, et al., Dietary advanced glycation end products are associated with an increased risk of breast cancer in Iranian adults, *BMC CANCER*, 2023, **23**.

O. O. Omofuma, L. L. Peterson, D. P. Turner, et al., Dietary advanced glycation end-products and mortality after breast cancer in the women's health initiative, *Cancer epidemiology, biomarkers & prevention*, 2021, **30**, 2217-2226.

S. Y. Kong, M. Takeuchi, H. Hyogo, et al., The association between glyceraldehyde-derived advanced glycation end-products and colorectal cancer risk, *Cancer Epidemiology, Biomarkers & Prevention*, 2015, **24**, 1855-1863.

Z. Mao, E. K. Aglago, Z. Zhao, et al., Dietary intake of advanced glycation end products (AGEs) and mortality among individuals with colorectal cancer, *Nutrients*, 2021, **13**, 4435.

Studies that did not report the relevant effect size

A. L. Mayén, E. K. Aglago, V. Knaze, et al., Dietary intake of advanced glycation endproducts and risk of hepatobiliary cancers: A multinational cohort study, *International journal of cancer*, 2021, **149**, 854-864.

Review studies

L. L. Peterson and J. L. Ligibel. Dietary and serum advanced glycation end-products and clinical outcomes in breast cancer, *BIOCHIMICA ET BIOPHYSICA ACTA-REVIEWS ON CANCER*, 2024, **1879**.

J.-i. Takino, K. Nagamine, T. Hori, A. Sakasai-Sakai and M. Takeuchi. Contribution of the toxic advanced glycation end-products-receptor axis in nonalcoholic steatohepatitis-related hepatocellular carcinoma, *World Journal of Hepatology*, 2015, **7**, 2459.
