# Supplementary material for: Dietary advanced glycation end‐products (dAGEs) are not associated with the risk of cancer incidence. A systematic review and meta‐analysis of prospective cohort studies
Source: Food Sci Nutr. 2024 Aug 11;12(10):7788–97. doi: 10.1002/fsn3.4396 (PMC11521677; doi:10.1002/fsn3.4396)
Supplement: Supplementary file 2 — Table S2. [file FSN3-12-7788-s002.docx]

**Supplementary tale 2: Certainty of evidence**

| **Certainty assessment** | | | | | | |  | | **Effect** | | **Certainty** | **Importance** |
| --- | --- | --- | --- | --- | --- | --- | --- | --- | --- | --- | --- | --- |
| **№ of studies** | **Study design** | **Risk of bias** | **Inconsistency** | **Indirectness** | **Imprecision** | **Other considerations** | **Number of participants** | **Number of cases** | **Relative (95% CI)** | **Absolute (95% CI)** |  |  |
| Cancer incidence | | | | | | | | | | | | |
| 5 | Cohort studies | very serious^a^ | not serious^b^ | not serious | not serious | none | 1220096 | 23229 | **RR 1.04** (0.94 to 1.15) | **1 more per 1,000** (from 1 fewer to 3 more) | ⨁⨁◯◯ Low | CRITICAL |

**CI:** confidence interval; **RR:** risk ratio

#### Explanations

a. There is a very serious risk of bias since two studies, which included 80% of the total participants, were rated as having a serious risk of bias. Additionally, the potential for residual confounding cannot be excluded. Downgraded.

b. Not serious inconsistency although I2=67.0%, which explained in sensitivity analyses based. Not Downgraded.
